# Referee acknowledgement for 2018

**DOI:** 10.1038/s41416-019-0490-x

**Published:** 2019-06-24

In this issue, we publish the names of those who reviewed the manuscripts for us in 2018. The Editor-in-Chief, Subject Editors and everyone involved in publishing *BJC* would like to extend our sincere thanks to the referees for contributing their expertise and time.

Daniel Abate-Daga

Omar Abdel-Rahman

Hinrich Abken

Eric O. Aboagye

Thomas Abrams

Rosalind Adam

Ari Adamy

Antoine Adenis

Pedro Aguiar Jr

Julio A. Aguirre-Ghiso

Jaffer Ajani

Lars Akslen

Tommy Alain

Donatella Aldinucci

Krasimira Aleksandrova

Laia Alemany

Roberta R. Alfieri

Alison L. Allan

James Allan

Victoria Allgar

Emma Allott

Luigi Aloj

Pierre Aman

Eitan Amir

Atieh Amouzegar

Pieter Anborgh

Annie Anderson

Victor Andrade

Nicolas André

Nadine Andrieu

Alan Anthoney

Lionel Apetoh

Natarajan Aravindan

Vincenzo Arena

Bruce Armstrong

Dirk Arnold

Melina Arnold

Roland Arnold

Rebecka Arnsrud Godtman

Gerhardt Attard

Riccardo Audisio

Dagfinn Aune

Anssi Auvinen

Gian Carlo Avanzi

Abraham Aviv

Hideo Baba

Zsuzsanna Bago-Horvath

Peter Bailey

Matthew Baker

Tejus Bale

Stuart Ballantyne

Rosemary Balleine

Alberto Ballestrero

Cynthia Bamdad

Aristotle Bamias

Udai Banerji

Pedro Barata

Ludovic Barault

Maria Barbolina

Carla Barceló

Fabrice Barlesi

Carlo Barone

Valeria Barresi

John Barrett

Gil Bar-Sela

Bristi Basu

Ken Batai

David Bates

Oliver Bathe

Luc Bauchet

Joshua Bauml

Daniel J. Becker

William Beck

Holger M. Becker

Philippe Bedard

Gundula Behrens

Tanios Bekaii-Saab

Deborah Belchis

Mattias Belting

Charlotte Benson

Angela Bentivegna

Laura Beretta

Christina Berg

Wendie A. Berg

Anna Berghoff

Johannes Berkhof

Pablo Berlanga

Eric Bernhard

Donald Berry

Francesco Bertolini

Allison Betof

Uddalak Bharadwaj

G. Jayarama Bhat

Roberto Bianco

Andreas Bikfalvi

Alan Bilsland

Romuald Binet

Andrea Biondi

Rebecca Birch

Tom Bird

Paolo Bironzo

D. Tim Bishop

Sarah Blagden

Jeremy Blaydes

Maria Blettner

Rune Blomhoff

Carl Blomqvist

Kevin Blyth

Kristien Boelaert

C. Richard Boland

Patrick Boland

Edwin Bolke

Elfriede Bollschweiler

Michael Bookman

Ivan Borbath

Sergio Bordel

Signe Borgquist

Josep Borrás

Claire Bosire

Patrick Bossuyt

Ioannis Boukovinas

Jean-Louis Boulay

Chiara Braconi

Andrew P. Bradford

Mary Sue Brady

Michael Braun

Jeremy Braybrooke

Donal Brennan

Paul Brennan

Darren Brenner

James Brenton

Kevin M. Brindle

Gero Brockhoff

Nigel Brockton

Jonathan Brody

Ewan Brown

Robert Brown

Jordi Bruna

Simon Buczacki

Ronald Bukowski

Sammuel Bunting

John Butler

Mary Butler

Reinhard Büttner

Maria Cabanillas

Yin Cai

Julien Calderaro

George Calin

Diego Calvisi

Anamaria Camargo

Carlos Camps

Erdem Canda

Francisco Jose Candido dos Reis

Ian Cannell

Yihai Cao

Maria Carlo

Richard Carvajal

Vincenzo Catalano

Emma Cattell

Guido Cavaletti

Iris Cervenka

Arlene Chan

David Chang

Lorraine Chantrill

Paul Chapman

Ian Chau

Mariana Chavez-MacGregor

Jeremy P. Cheadle

Srikumar Chellappan

Alice Chen

Ching-Chow Chen

Eric Chen

Gang Chen

Ann-Lii Cheng

Bin Cheng

Edwin Chong Wing Cheung

Wisit Cheungpasitporn

Ferdinando Chiaradonna

Melvin Chin

Gabriela Chiorean

Shubhada Chiplunkar

Minsig Choi

Irene Chong

Alan Christie

Liz Christie

Heather Christofk

Richard Christopherson

David Church

Jane Churpek

Walter Chwals

Joseph Ciccolini

Callisia N. Clarke

Hans Clevers

Gary Clifford

David E. Cohn

Rovel Colaco

Graham Colditz

Helen Coleman

Andrew Collier

Peter Collins

Jean-Damien Combes

Paolo Comoglio

John Conibear

Claire Connell

Susie Cooke

Colin S. Cooper

Pippa Corrie

Nadia L. Costa

Leopoldo Costarelli

Michele Cote

Paul Cottu

Jennifer Couch

Amanda Coutts

Alan Craft

Adrian Crellin

Chiara Cremolini

Nicola Cresti

Margaret Cruickshank

Carien Crutzberg

Ilona Csizmadi

Michele Cummings

Giuseppe Curigliano

Joseph Curry

Anne Cust

Anneleen Daemen

Nergiz Dagoglu

Kristina Dahlstrom

Piero Dalerba

Angus Dalgleish

Arnaldo D’Amico

Adam Dangoor

Sahar Aly Daoud

Altaf A. Dar

Sarah Darby

Sunit Das

Sarah E. Daugherty

Diwakar Davar

Jeff Davies

Ian Davis

Chi-Ping Day

Zandra Deans

Waldemar Debinski

Johann DeBono

Thomas Decaens

Julie Decock

Shoukat Dedhar

Miriam Deeny

Ugo De Giorgi

Nienke De Glas

Amy C. Degnim

Paolo Dellabona

Marzia Del Re

Cosimo De Nunzio

Alban Denys

Assunta De Rienzo

Juan Pablo De Rivero Vaccari

Tessa De Vrieze

Mahua Dey

Nandini Dey

Tony Dhillon

Robert Diasio

Mario Dicato

Paul Dickman

Christina Dieli-Conwright

Palma Dileo

Richard Dillon

Marie-Therese Dimanche-Boitrel

Charles Dimitroff

Maurizio D’Incalci

Luc Yves Dirix

Dolores Di Vizio

Eytan Domany

Balazs Döme

Timothy Donahue

W. Alexander Donald

Kim Donghee

Frede Donskov

Isabel Dos Santos Silva

Patrick Dougherty

Crislyn D’Souza-Schorey

Catherine Dube

Steven DuBois

Eric Duell

Stephen W. Duffy

Janice P. Dutcher

Mark Duxbury

Martin Dyer

Lars Dyrskjot

Paul J. Dyson

Helena Earl

Richard Edmondson

Joanne Edwards

Alexey Efanov

Alexander Eggermont

Susumu Eguchi

Anders Ekbom

Yasser Elborai

Imane El-Dika

K. Miriam Elfström

Kevin M. Elias

Heather Eliassen

Areej El-Jawahri

Matthew Ellis

Cathy Eng

Andrew S. Epstein

David Epstein

Bircan Erbas

Mikael Eriksson

Ferry A.L.M. Eskens

Manel Esteller

Arash Etemadi

Alan Evans

D. Gareth Evans

Tom Fahey

Benjamin Fairfax

Stephen Falk

Mohammad Fallahi-Sichani

Bishoy Morris Faltas

Olusola Faluyi

Alberto Farolfi

Matteo Fassan

Steven Feldman

Enriqueta Felip Font

Dyke Ferber

Sofia Fernandes

Napoleone Ferrara

Maria Feychting

William Figg

Barbara Fingleton

Jonathan L. Finlay

Michael A. Firer

Aude Flechon

Elizabeth Folkerd

Gunnar Folprecht

Simona Fontana

Lorenzo Fornaro

Matthew Forshaw

Renée Fortner

Steven Foster

Floyd J. Fowler

Douglas Fraker

Callum Fraser

Claudia Fredolini

Heinz Freisling

Carlo Fremd

Claire Friedemann-Smith

Daichi Fujimoto

Yohei Funakoshi

Marike Gabrielson

David K. Gaffney

Justin Gainor

Timo Gaiser

Christopher Gallagher

Javier Gallego Plazas

Eve Gallop-Evans

Matthew Galsky

Ram Ganapathi

Shridar Ganesen

Apar Kishor Ganti

Montserrat Garcia-Closas

Custodia Garcia-Jimenez

Dale Garsed

Zoran Gatalica

Constantine A. Gatsonis

Poonam Gautam

Andrew Gaya

Christoffer Gebhardt

Jeanine Genkinger

Angela George

Marco Gerlinger

Grigoris T. Gerotziafas

Francois Ghiringhelli

Alireza Ghoreifi

Giuseppe Giaccone

Marios Giannakis

Raffaella Giavazzi

Duncan Gilbert

Anthony Gill

Robert Gillies

Roopinder Gillmore

Silvia Giordano

Edward Giovannucci

Martin E. Gleave

Paul Glen

Bengt Glimelius

L. Michael Glode

Robert Glynee-Jones

Michael Gnant

Colin Goding

James J. Going

Jakub Golab

David Goldgar

Aron Goldhirsch

Simon Gollins

Andrea Gombos

Carlos Gomez-Roca

Zhihong Gong

Mohammad Goordazi

Kylie Gorringe

Charlie Gourley

Amandine Gouverneur

Olivier Govaere

Rondell Graham

Joe Gray

Godfrey Grech

John E. Greenlee

Alastair Greystoke

Alexandru Dan Grigore

Anita Grigoriadis

Anita Grigoridis

Naomi Gronich

Guowei Gu

Jian Gu

Fiorella Guadagni

Chantal Guillemette

Fernando Guimarães

Jennifer Gunter

Jianping Guo

Kun Guo

Salman Yousuf Guraya

Vsevolod Gurevich

Barry A. Gusterson

Laurel Habel

Sassan Hafizi

Jaana Hagström

Niels Halama

Lindsay Hall

Yasuo Hamamoto

William Hamilton

Chaofeng Han

Karen Piper Hanley

Gerry Hanna

Aaron Hansen

James Harding

Ran Harel

Adrian Harris

Ewen Harrison

Mark Harrison

Takaaki Hasegawa

Mathieu Hatt

Matthew Hatton

Yoku Hawakawa

Maria Hawkins

Tamara Haygood

Valtteri Häyry

Sirpa Heinävaara

C. Simon Herrington

Christopher Hewitt

Ian Hickson

Geoff Higgins

Howard Hochster

Aaron Hoffman

Paul Hofman

Pernille Højman

Antoine Hollebecque

Andrew J. Holmes

Wanjin Hong

Carmen H. Houben

Pingzhao Hu

Cai Huang

Chen Huang

Raymond Y. Huang

Shile Huang

Yi Huang

Robert Huddart

Ivo Huijbers

Jan-Jong Hung

Dezheng Huo

Sara Hurvitz

Hisham Hussan

David Hyman

Mohammad Ilyas

Stefano Indraccolo

Peter F. Infante

Federico Innocenti

Satoshi Inoue

Monica Iorfida

Kelly E. Irwin

Meredith Irwin

Steven Isakoff

Hiroki Ishii

Mitsuru Ishizuka

Alexander Ishov

Takahiro Ito

Tim Iveson

Rene Jackstadt

Ira Jacobs

Rakesh Jain

Anders Jakobsen

Ted James

Nigel Jamieson

Filip Janku

Tobias Janowitz

Marnix Jansen

Sarah Jefferies

Mazda Jenab

Allan Jensen

Lars Henrik Jensen

Carmen Jerónimo

Minghong Jiang

Markus Joerger

Jonathan Joffe

Adam Jona

Lee Jones

Robert Jones

Robin Jones

V. Craig Jordan

Claus Jorgensen

Medha Joshi

Ian Judson

Sushant Kachhap

Luna Kadouri

Christoph Kahlert

Kyoichi Kaira

Ersan Kalay

Halime Kalkavan

Tania Kalsi

Gyeong Hoon Kang

Hee-Eun Kang

Amalia Karahalios

Giorgos Karakousis

Eva Karamitopoulou

Solon Karapanagiotis

Ioannis Karydis

Jakob Kather

Joonas Kauppila

Lital Keinan-Boker

Charles Kelly

Oliver Kepp

David Kerr

David Kessel

Saadi Khochbin

Alok Khorana

Adam S. Kibel

Nam Kyu Kim

Won Kim

Randall J. Kimple

Tari King

Hideaki Kinugasa

Henry C. Kitchener

Jennifer Knox

Martin Köbel

Ernest Kohorn

E. Anders Kolb

Chihaya Koriyama

Antonis E. Koromilas

Siran M. Koroukian

Richard L Kradin

Oliver Krämer

Daniel Krappman

Jürgen Krauss

Matthew Krebs

Malathi Krishnamurthy

Glen Kristiansen

Mary E. Kroll

Satish Kumar

Yukinori Kurokawa

Ozgur Kutuk

Michael Ladomery

Anne-Vibeke Lænkholm

Sonia Lain

Sunil Lakhani

Clara J.K. Lam

Angela Lamarca

Diether Lambrechts

Philip Lammers

Carol Lange

Ruth Langley

Cord Langner

James Larkin

Pierre Laurent-Puig

Martin Lavin

Michael Leahy

Valerie LeBleu

Estelle Leclerc

Agnes Lee

Jonathan M. Lee

Jun Hee Lee

Michael Lee

Stefan Leger

Natasha Leighl

Mario Leitao

Julie Lemieux

Benedicte Lenoir

Robert Leonard

John Le Quesne

Enrique Lerma

Christophe Le Tourneau

Douglas Levine

Chong Li

Daneng Li

Qingguo Li

Wei Li

Xiujuan Li

Yan Li

Zhenfei Li

Stuart M. Lichtman

Christopher Lieu

Noralane M. Lindor

Colin Lindsay

Linda Lindström

Joachim Lingner

Bodo Lippitz

Esther Lips

Karin List

Ann-Margaret Little

Bolin Liu

Delong Liu

Ying Liu

Zheng-Gang Liu

Christopher Lo

Regina Lo

Magnus Løberg

Fiona Lofts

Matthias Löhr

Martijn Lolkema

Daniel Longley

Marcio Lopes

Juanita Lopez

Florian Lordick

James B. Lorens

Michael Lotze

Fotios Loupakis

Maeve Lowery

Xiaojie Lu

John Lunec

Philip Lupo

William Lydiatt

David J. Lynn

Wen We Ma

Yuk Ting Ma

Teresa Macarulla

Una Macleod

Iain Macpherson

Ayman Madi

Mary Beth Madonna

Markus Maeurer

James G. Mainprize

Rob Mairs

Tom Maishman

Si Ming Man

Mahitosh Mandal

Sendurai Mani

Alberto Mantovani

Victoria Mar

Anthony Maraveyas

Fabio Marchi

Stefan Marciniak

Catherine Marinac

Maurie Markman

Deborah J. Marsh

Aileen Marshall

Lesley-Ann Martin

Richard Martin

Gerard Mascara

Luca Mastracci

Athena Matakidou

Ewy Mathe

Andreia Matos

Hiroyuki Matsubayashi

Koji Matsumoto

Kunio Matsumoto

Katherine Matthay

Patrick Matthias

Tim Maughan

Joan Maurel

Marcela Maus

Felicity May

Vincenzo Mazzaferro

Katherine McAllister

Jessica N. McAlpine

Joshua McCarroll

Sarah McClelland

Keith McCrae

Ultan McDermott

Bradley McGregor

Pamela McKay

Heather Mckinnon

Una McMenamin

Donald McMilllan

Richard McNally

Keely McNamara

Mairead McNamara

Margaret L. McNeely

Alan McNeill

Iain McNeish

Grant McQuaker

Jean Paul Medema

Ranee Mehra

Babak J. Mehrara

Ignacio Melero

Eline Menu

Didier Meulendijks

Nadav Michaan

Dominique Michaud

Judith Michels

David Miles

Lucia Miligi

Anthony B. Miller

William R. Miller

Ian G. Mills

Anna Minchom

Lisa Mirabello

Haitham Mirghani

Alexander Mirnezami

Karuna Mittal

Hisamitsu Miyaaki

Simone Mocellin

Helmout Modjtahedi

Siver Moestue

Carolin Mogler

Damian Mole

Richard Molloy

Marie E. Monaco

Silvio Monfardini

Gisele Monteiro

Michael Montemurro

Gil Mor

Carlos S. Moreno

Victor Moreno

Carys Morgan

David Morris

Simone Mörtl

Susan Moug

Colin Muirhead

Somnath Mukherjee

Paul B. Mullan

William Mullen

Ramon Munoz-Chapuli

Elisabetta Munzone

Satoshi Murata

Peter Murchie

Neil Murphy

Graeme Murray

Giorgio Mustacchi

Manabu Muto

Jeffrey N. Myers

Tor Myklebust

Rosa Nadal Rios

Silvio Nadalin

Margherita Nannini

Gerardo Nardone

Steven A. Narod

Mohan Natarajan

Atsushi Natsume

Jean-Charles Nault

Angel R. Nebreda

Albrecht Neesse

Naveen Neradugomma

Carola Neumann

Kimmie Ng

Andrea Nicolini

Yuanjie Niu

Simon Noble

Nicola Normanno

Jonathan Nowak

Stefan Nowicki

Lennarth Nystrom

Mary E.R. O’Brien

Takahiro Ochiya

James P.B. O’Connor

Peter H. O’Donnell

Steffi Oesterreich

Judith Offman

Shuji Ogino

Jason Oke

Ken A. Olaussen

David Olmos

Jason Ong

Wee Loon Ong

Christiane Opitz

Elisa Oricchio

Mai-Britt Ørntoft

Noelle O’Rourke

Nicolas Orsi

Juan Oscar

Quinn Ostrom

Ify Osunkwo

Jose Palacios

Viktoria-Varvara Palla

Claire Palles

Giuseppe Palmieri

Hardev S. Pandha

Gladell Paner

Lisa Y. Pang

Klaus Pantel

Andrea Papadia

Efthymia Papaevangelou

Thales Papagiannakopoulos

James Park

Sue K. Park

Viktoriya Paroder

Patrizia Pasanisi

Alessandro Passardi

Antonio Passaro

Scott Patterson

Jim Paul

Patricia Pautier

Edward Pavlik

Miranda Payne

Karl Peggs

Uwe Pelzer

George Pentheroudakis

Cátia S. Pereira

Nathan Perlis

Pierpaolo Peruzzi

Cheryl E. Peters

Godefridus Peters

Max Petrov

Russell Petty

Paul Pharoah

Martin Pichler

Filippo Pietrantonio

Dietmar Pils

Matthias Pinter

Meredith Pittman

Todd Pitts

Elizabeth Plummer

Jan Poleszczuk

Michael Pollak

Patrizia Pontisso

Emilio Porfiri

Johanneke Portielje

Sophie Postel-Vinay

Chantevy Pou

Pataje G.S. Prasanna

Jochen Prehn

Pat Price

Timothy Price

Kathleen I. Pritchard

Andrea Prota

Elena Provenzano

David Pryce

Eero Pukkala

Cornelis J.A. Punt

Colin Purdie

Lisha Qi

Bin-Zhi Qian

Fuming Qiu

Federico Quaini

Taihao Quan

James Rae

Daniel Raftery

Arman Rahman

Christy Ralph

Francisco X. Real

Andrew Redfern

H Paul Redmond

Nicholas Reed

Christoph Reissfelder

Jessica Reynolds

Ricardo Ribeiro

Frances Richards

Kyle A. Richards

Jennifer Richer

Isidore Rigoutsos

Alistair E. Ring

Brian Rini

Susan Rittling

Victor M. Rivera

Stephane Rocchi

Claus Rödel

Alexander Roelle

Cécile M. Ronckers

Carlos Rosales

Rafael Rosell

Paul J. Ross

Arnaud Roth

Andrew Rowland

Patricia Roxburgh

Campbell Roxburgh

Sougata Roy Chowdhury

Greg Rubin

Antonio Russo

Francesco Paolo Russo

Elizabeth Ryan

Renaud Sabatier

Sharanjot Saini

Koichi Sakakura

Gianluca Sala

Ramon Salazar

Ahmed Salem

Federica Saletta

Leonard Saltz

Rajeev S. Samant

Leslie Samuel

Owen Sansom

Marta Santisteban

Cristina Santos

Pablo Sarobe

Carol A. Sartorius

Hiroki Sasaki

Niranjan J. Sathianathen

Toshiro Sato

Mark Saunders

Elinor Sawyer

Aldo Scarpa

Matt Scarpelli

Mario Scartozzi

Erik Schadde

Birgit Schilling

Stefano Schipani

Gudrun Schleiermacher

John Schmitz

Hans-Joachim Schmoll

Martin Schneider

Minouk Schoemaker

Marij Schoenmaekers-Welters

Patrick Schöffski

Zachary Schug

Anna Schuh

Joachim Schüz

Judith Ann Schwartzbaum

Heidi Schwarzenbach

Daniel Sciubba

Giuseppe Sconocchia

David Scott

Paul J. Scotting

Cynthia Sears

Andrew Seidman

Jenny Seligman

Stefano Serra

Vijayasaradhi Setaluri

Einat Shacham-Shmueli

Kelly M. Shaffer

Payal D. Shah

Jonathan Shamash

Dipali Sharma

Rohini Sharma

Paul Shaw

David Sheehan

Keiran Sheahan

Anthony F. Shields

Dong Wook Shin

Seokmin Shin

Yusuke Shiozawa

Keisuke Shirai

Ke Shuai

Michael Shurin

Daniela Sia

D. Robert Siemens

João Silva

Robert H. Silverman

Kate Simms

M. Celeste Simon

Jennifer Sims-Mourtada

Ka Tat Siu

Ondrej Slaby

Age K. Smilde

Grace Smith

Samuel Smith

Elizabeth Smyth

Petur Snaebjornsson

Adam Snook

Kenzo Soejima

Payaningal Somanath

Mingyang Song

Amir Sonnenblick

Anil Sood

Halfdan Sorbye

John Souglakos

Nuno Sousa

Melanie Spears

Ian Spendlove

James Spicer

Daniel E. Spratt

Pugazhendhi Srinivasan

Amitabh Srivastava

Adrian Stanley

Margaret Stanley

Martin Stanulla

Lesley Stark

Naureen Starling

Justin Stebbing

Nicola Steele

Jennifer Stein

Stacey Stein

Arnulf Stenzl

Lindsay Stetson

Victoria Stevens

Sheila Stewart

Sebastian Stintzing

Jon Stobo

Julie A. Stone

Michael Stotz

Michael B. Streiff

Sophia Subat

Kenichi Suda

Haruo Sugiyama

Lillian L. Sui

Ryan Sullivan

Jing Sun

Xueying Sun

Yin Sun

Karin Sundström

Claudiu Supuran

Mario Suva

Pawel Swietach

Matt Sydes

Stefan Symeonides

R Paul Symonds

Gergely Szakács

Martina Taborelli

Ayumu Taguchi

Julien Taieb

Diana Tait

Kazuaki Takabe

Toru Takano

James Talmadge

Teruko Tamura-Niemann

Hiroaki Tanaka

Sarah K. Tasian

A. Malcolm Taylor

Peter M. Taylor

Sian Taylor-Phillips

Daniel Tennant

Krishnansu Tewari

Fiona Thomson

Aaron Thrift

Wayne Tilley

John F. Timms

Nick Timpson

Anna Tinker

Ellie Tiplady

Amit K. Tiwari

Antonella Tomassetti

Ian P.M. Tomlinson

Adetunji Toriola

Davis Y. Torrejon

Jan Tost

Cecilie Totland

Yann Touchefeu

Rhian Touyz

Sophie Trefely

Inaki F. Troconiz

Jone Trovik

Claudia Trudel-Fitzgerald

Konstantinos Tsilidis

Shoichiro Tsugane

Shi-Ming Tu

Clare Turnbull

Andrew Tutt

Chris Twelves

Rudolf Übelhart

Stefano Ugel

Paola Ulivi

Juliet Usher-Smith

Dominique Valteau-Couanet

Claudia Valverde

Mark Van Berge Henegouwen

Carla H. Van Gils

Bethany Van Guelpen

Mieke Van Hemelrijck

Han Van Krieken

Ashok Venkitaraman

Balaji Venugopal

Louis Vermeulen

Jan B. Vermorken

Pamela Vernocchi

Emily Vertosick

Bruno Vincenzi

Amaya Viros

Arndt Vogel

Dan Toby Vogl

Johannes Vom Berg

Michael S. Von Bergwelt-Baildon

Tom Waddell

Jon Wadsley

Uwe Wagner

Reema Wahdan-Alaswad

Zev Wainberg

Michael Wakelam

Jerome Waldispuhl

Alex Walker

Matthew Wallis

Fiona Walter

Harriet Walter

Li Lily Wang

Chih-Hong Wang

Cuiyan Wang

Michael Wang

Peng-Hui Wang

Zhiwei Wang

Andrea Wang-Gillam

Justin Waters

Ashani Weeraratna

Esther K. Wei

Zhang Weihua

Alan Wells

Claire Wells

Michael Welsh

Martin Werner

Maria Werner-Wasik

Jelle Wesseling

Nick West

Christoph Westphalen

Yvonne Wettergren

Jessica Whitburn

Mark R. Wick

Martin Widschwendter

Stephen J. Wigmore

Karn Wijarnpreecha

Brigitte Wilczek

Ania Wilczynska

Gareth Haydn Williams

Reinhard Windhager

Boris Winterhoff

Penella J. Woll

Barbara Wollenberg

Deborah J.L. Wong

Grace Wong

Reen Wu Winson Wong

David Wynford-Thomas

Jingwu Xie

Rui-hua Xu

Krishna Yalla

Tesshi Yamada

Hiroko Yamashita

Shi-Ming Yang

Zhi-Zhang Yang

Masakazu Yashiro

Melinda S. Yates

Lucy Yates

Iwei Yeh

Rinat Yerushalmi

Hye Jin You

Caroline Young

Robin Young

George Yousef

Jianhua Yu

Jun Yu

Kenneth Yu

Yaozong Yuan

Kyuson Yun

Paul Zarogoulidis

Anne Zeleniuch-Jacquotte

Jianmin Zhang

Rugang Zhang

Xianlan Zhang

Yun-Ling Zheng

Hong Zhou

Danna B. Zimmer

Wolfgang Zimmermann

Laurence Zitvogel

Gabriele Zoppoli

Petra Zurbig

